# Collaborative Distributed Scheduling Approaches for Wireless Sensor Network

**DOI:** 10.3390/s91008007

**Published:** 2009-10-13

**Authors:** Jianjun Niu, Zhidong Deng

**Affiliations:** 1 State Key Laboratory of Intelligent Technology and Systems, Tsinghua National Laboratory for Information Science and Technology, Department of Computer Science and Technology, Tsinghua University, Beijing 100084, China; 2 Beijing Special Engineering Design and Research Institute, Beijing 100028, China; E-Mail: njj05@mails.tsinghua.edu.cn

**Keywords:** wireless sensor network, energy, scheduling, collaboration, distribution

## Abstract

Energy constraints restrict the lifetime of wireless sensor networks (WSNs) with battery-powered nodes, which poses great challenges for their large scale application. In this paper, we propose a family of collaborative distributed scheduling approaches (CDSAs) based on the Markov process to reduce the energy consumption of a WSN. The family of CDSAs comprises of two approaches: a one-step collaborative distributed approach and a two-step collaborative distributed approach. The approaches enable nodes to learn the behavior information of its environment collaboratively and integrate sleep scheduling with transmission scheduling to reduce the energy consumption. We analyze the adaptability and practicality features of the CDSAs. The simulation results show that the two proposed approaches can effectively reduce nodes' energy consumption. Some other characteristics of the CDSAs like buffer occupation and packet delay are also analyzed in this paper. We evaluate CDSAs extensively on a 15-node WSN testbed. The test results show that the CDSAs conserve the energy effectively and are feasible for real WSNs.

## 1. Introduction

Wireless Sensor Networks (WSNs) have attracted increasing interest in recent years [1]. Normally, a WSN consists of a very large number of wireless network nodes integrating many capacities like sensing, computation and communication. In order to monitor a field of interest, these WSN nodes collect data from its surrounding environment and transport the data to the sink node collaboratively via multi-hop communication. WSNs have been successfully applied to structural health monitoring [2], localization of an object [3], breeding behavior on Great Duck Island [4] and many other application domains. Their wide application implies their potential future in various fields. However, WSNs are typically characterized by constrained energy due to their energy recharging difficulty in many applications. This feature makes them more challenging in real applications and needs further research efforts.

In this paper, we consider a series of applications of WSNs, where the nodes are all stationary. All of these nodes sample their environment information simultaneously and periodically, and then transmit the data to a sink node through multi-hop communication. These applications could cover structural health monitoring, location of objects and other applications. The WSN in this paper is based on a contention network, whose use has been increasing in popularity. Normally, in a contention network idle listening wastes most of the energy [1,5]. Packet retransmission also wastes energy because it not only consumes much energy on retransmission, but also makes back-off times longer, which results in much more energy being wasted in the idle state. We propose herein a family of collaborative distributed scheduling approaches (CDSAs) for the purpose of reducing the energy consumption of WSNs, which are based on the discrete time Markov chain (DTMC). The CDSAs enable nodes to learn the behavior of their environment, and estimate the working features of their child nodes and parent node through collaboration. The CDSAs integrate sleep scheduling with packet transmission scheduling together to reduce energy consumption. Only when a node estimates that all its child nodes are not transmitting packets and its parent node can successfully receive its data packet, it schedules transmission of its data packet to its parent node. It can also schedule itself to a sleep state when it has transmitted all its data packets or when the serious collisions exist. We analyze the adaptability and practicability of the CDSAs. We also validate the CDSAs based on the CSMA/CA protocol through simulation and evaluate them on a WSN testbed. The results show that the proposed approaches can save energy effectively.

The rest of this paper is organized as follows. In Section 2, we describe the background and elaborate our contributions. In Section 3, we present the network model. In Section 4, we present the family of CDSAs, describe the one-step collaborative distributed approach (O-CDSA) and the two-step collaborative distributed approach (T-CDSA) in detail. In Section 5, the adaptability and practicality of the two approaches are discussed. In Section 6, some experiments are carried out to evaluate the performances of CDSAs, and the experimental results are further discussed. In the last section, we conclude this paper and suggest some future research topics.

## 2. Background

Generally a WSN node can be put into one of the following five states: Transmit, Receive, Idle, Sleep and Sense. Each state corresponds to a specific power consumption level. Energy consumption in Sense state is relevant to a specific application, while energy consumptions in other states are related to the work process of a node. In addition, energy consumption for the radio component in a node is much greater than that of other components in the majority of WSN applications [6]. Therefore, the energy consumption in the Sense state is ignored in this paper. In terms of energy consumption per time unit, the Transmit state usually consumes the most energy, Receive consumes second to Transmission, then is Idle and Sleep state consumes the least among these four states. Therefore a common technical solution to prolong network lifetime is to schedule nodes to pending most working time in the states with lower energy consumption, and research on scheduling mechanisms has become and increasingly hot topic in the field of WSNs. Reference [7] summarized a lot of distributed scheduling mechanisms designed for WSNs. However, more and more distributed scheduling approaches to improve energy efficiency have been proposed in recent years. The main objective of scheduling mechanism is to reduce energy consumption and prolong the lifetime of WSNs, which is the fundamental function of power management plane related to every layer in the protocol architecture [8].

Many papers study issues on scheduling mechanisms in MAC layer to minimize energy consumption [9-16]. Some of them have focused on the conflict-free networks like TDMA network or CDMA network [9-13] to save energy by scheduling nodes to work in their own time slot and change to sleep state when nodes are out of their own time slot. Others focused on the contention-based networks [14-16]. Normally, TDMA-based MAC scheduling mechanisms can guarantee delay bounds due to collision elimination, while contention-based MAC scheduling mechanisms may result in non-deterministic packet delays due to the collisions and the packet retransmissions. However, the contention-based MAC protocols are not only robust to the network topology and network load, but also are scalable to network size and node density. There are many other scheduling approaches to study energy saving above MAC layer [17-21]. Reference [18] presented an energy-efficient organization method, which schedules the nodes to sleep by sensing the object location collaboratively, and to perform probability awakening in a distributed manner. Reference [19] proposed a sensor scheduling approach, jointing sensing coverage and network connectivity, to save energy in the absence of accurate location information. The authors of [20] presented a scheduling algorithm which allows nodes to learn the shape of the traffic through observing upstream neighboring nodes independently, and then shape the transmission traffic and determine its sleep time for saving energy. The authors in [21] proposed a wake-up scheduling algorithm that determines the wake-up frequency of the nodes. Because scheduling approaches above the MAC layer are not designed for a specific MAC protocol, they can be easily used in many applications. Most scheduling approaches reduce energy consumption by increasing the sleep time of nodes. Moreover, in a contention-based network, some energy wastes on the packets transmission. Therefore some papers, like [14] and [20], also took packet transmission scheduling into consideration.

In a WSN, which is based on a contention channel and samples environmental information simultaneously and periodically, nodes normally initiate data packet transmission simultaneously, which leads to energy waste due to the serious collision and idle listening. For example, a structural health monitoring system should correctly collect raw data information on structural behavior in a continuous and periodical way. These data collected from each site are used by structural engineers to make the modal analysis and study the structural properties. Focusing on this type of WSN application, we propose a family of CDSAs. Our CDSAs are now studied above the MAC layer and we assume that the contention-based MAC protocol exists underneath. The CDSAs can also be applied to other applications based on contention channel, because our CDSAs are based on DTMC and the scheduling probability parameters are all from the statistical value.

From the articles we have researched, our CDSAs are uniquely different from all scheduling approaches used by other papers. On the one hand, we set up working state model based on DTMC to schedule nodes' sleep and data transmission, on the other hand, we also construct the behavior of the child nodes, the sleep behavior of the parent node and the dynamics of communication channel to effectively schedule nodes' state. The behavior of the child nodes is inspired by the analytical model of the next-hops in the paper [22], but our behavior model of the child-nodes is based on the CSMA/CA protocol and is implemented through statistical methods. Moreover, the behavior of neighboring nodes and state information of communication channel are new in this paper and are learned from another Markov process model. From a higher view above the MAC layer, the transmission scheduling of CDSAs can adjust the transmission rate of nodes, which is somewhat similar to the rate control transport algorithm in [23,24] and the application data unit download scheduling in paper [25]. But the approaches in the above are centralized and base station is required to make a decision. Our CDSAs are distributed and can be applied to each node. Our basic purpose is to let nodes collaboratively learn the scheduling probability parameters for the working state scheduling. We also discuss the adaptability and practicability of the proposed two CDSAs in this paper. We evaluate the CDSAs with the underlying CSMA/CA protocol and discuss some network performances of the CDSAs. The results show that these two approaches can reduce energy consumption effectively. Moreover, the O-CDSA is practicable to a lightweight workload application and T-CDSA can also be applied to heavyweight workload application.

## 3. Network Model

**Figure 1.**
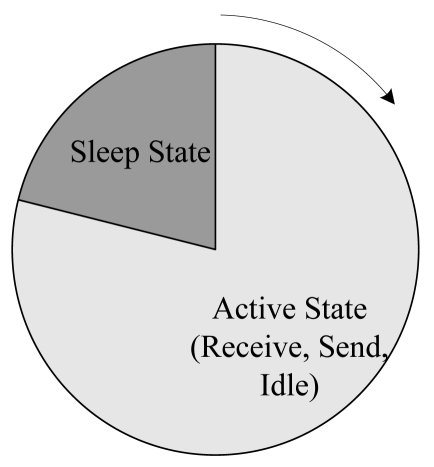
Work cycle of a node.

In this paper we consider a series of WSN applications, which collect information periodically and simultaneously, and transport the data as packets to a sink node. Certainly there is no sink node number limitation, but we adopt only one sink node in this paper. Each node in the system is equipped with an omni-antenna. The data transmission and receiving are two separate processes and cannot work simultaneously. A node can work in one of four basic states: Receive (R), Transmit (T), Idle (I) and Sleep (S). The work cycle of a node is the time period from the last wake-up time to the next wake-up time. The work process is illustrated in Figure 1. When a node wakes up, it will be in the Active state. It can receive data, send data to its next-hop or stay in an Idle state. When it enters Sleep state, it does not respond to the environment until the next wake-up time. When it wakes up, it will start a new work cycle. In this paper, we set the same wake-up time for all nodes at a fixed time interval. Given its heavy work load or the busy channel, a node may not fall to sleep in a cycle. Each node simultaneously produces a fixed-size packet at a regular interval. And after a node receives the data from itself or from other nodes, it will store them in a first-in-first-out (FIFO) queue before it sends them out.

**Figure 2.**
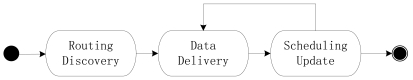
System running process.

The communication channel is a shared single channel. It is assumed that the some contention-based MAC protocol is applied, probably the CSMA/CA protocol [26]. We consider a stationary WSN and adopt a static route strategy for it. A node has only one next-hop node in its routing table. For any route in whichever follows, upstream refers to the direction which data packets are transmitted to the sink node. And in the downstream, the data packets are transmitted from the sink node. We call node A parent node of node B when node A is the next-hop in upstream of node B. And node B is the child node of node A. The static route is formed at the routing discovery (RD) stage at each experiment, as is shown in Figure 2.

A network-wide time synchronization protocol is assumed to ensure that all of nodes will have the same wake-up time. However, the time accuracy of our CDSAs is not as strict as those of TDMA based approaches because the work cycle is much longer than a time slot.

## 4. Collaborative Distributed Scheduling Approach

### 4.1. Overview of CDSAs

There are two separate stages for the family of CDSAs. These stages are: data delivery (DD) stage and scheduling update (SU) stage. At the DD stage, the main tasks of nodes are elaborated as follows:

1. All nodes have the same awakening time at a fixed time interval. We focus on a series of WSN applications collecting information periodically and simultaneously. Therefore, all nodes have the same awaking time at a fixed time interval, and transmit the data to a sink node through multi-hop communication afterwards.
2. All nodes simultaneously generate their original fix-sized data packet at a regular interval.
3. Each node decides independently whether it should transmit its data packet according to its scheduling probability. If permitted, it will send out its data packets to the next-hop. Otherwise, it will change to Idle state. If there is no other data packets received when the node is in Idle state, the node will begin a new scheduling process after a while.
4. If the sender does not receive the ACK frame in a specific time period, it must re-transmit the data packet according to its scheduling probability.
5. After a node has sent out all data packets in its FIFO queue, it will decide whether it goes to sleep or not according to its scheduling probability.
6. The nodes, being in Sleep state, will wake up at the next wake-up time. For the nodes that are still in active phase will continue its operation when the next work cycle starts.
7. The MAC protocol implements the transmission and reception processes underneath.
8. Each node sums up the number of data packets that have been successfully transmitted, the number of packets transmitted, the total transmission time and the total active time. These four parameters are exchanged at the SU stage.

At the SU stage, some of the recorded statistical variables are exchanged among adjacent nodes to help determine the scheduling matrix (SM) of each node. Then the SM is used to schedule node states at the next DD stage. The DD stage and the SU stage compose a round of the CDSAs. And the system will be running rounds of CDSAs. According to the different scheduling update mechanism, we put forward the following two CDSAs to collaboratively calculate the SM.

### 4.2. One-Step Collaborative Distributed Scheduling Approach

The reason for calling this approach a one-step collaborative distributed scheduling approach is because only one step — transmitting statistical variables to upstream — is required in the exchange process at the SU stage.

#### 4.2.1. Scheduling Process

**Figure 3.**
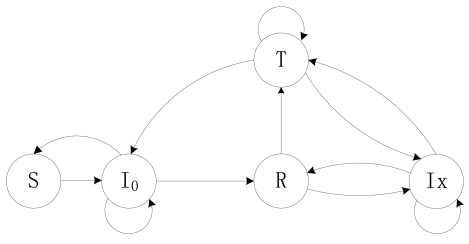
State transition diagram of a node in O-CDSA. where I_0_ represents the Idle state with empty buffer, Ix is the Idle state with its FIFO queue not empty, S, R and T represent the states of Sleep, Receive and Transmit respectively.

For O-CDSA, we design that a node does not go to sleep until it has sent out all its data packets in its FIFO queue. We divide the Idle state into I_0_ and Ix states. I_0_ refers to the Idle state with an empty FIFO and Ix refers to the Idle state with its FIFO queue not empty. At the DD stage, the working state model of a single node can be described as a DTMC model, which is illustrated in Figure 3. After waking up, a node stays in an I_0_ state. After it receives a data packet, it may change to Ix state or begin to transmit a data packet from its FIFO queue according to its scheduling probability. If it fails to transmit the data packet, it will re-transmit the data packet again or shift to Ix state. It either changes to I_0_/Ix state or re-transmits another data packet from FIFO queue again after it has successfully transmitted a packet. After having transmitted all data packets in its FIFO queue, it may either shift to its Sleep state, or stay in I_0_ state and then begin scheduling state again for a while. If a node goes to Sleep, it will remain this state until the next wake-up time.

**Figure 4.**
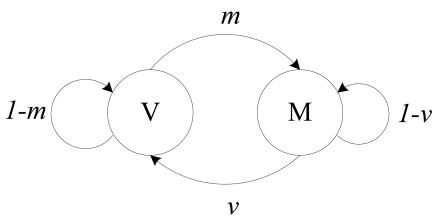
State transition of child nodes. where the transition probability *v* is the probability of at least one of its child nodes changed to Transmit state, and *m* is the transition probability from state V to state M.

The transmissions of child nodes influence their parent node. Any transmitting of the child nodes will conflict with the transmitting process of their parent node. Thus, in order to schedule a node's state effectively, the node should correctly estimate the states of its child nodes. We introduce a behavior model of child nodes with two states: Mute (M) and Viral (V), indicating the effects of the child nodes on their parent node. The state M corresponds to all its child nodes not in Transmit state, while the state V corresponds to at least one of its child node in Transmit state. Therefore, a node can send out its data packets only when the behavior model of its child nodes is in M state. Transitions between M and V are illustrated in Figure 4.

In order to transmit packets successfully, a node must compete with other nodes close to it and data packets always conflict with each other. Thus, in order to schedule a node's state effectively, the node should also learn how busy the communication channel is. We introduce a parameter *β*, which indicates the ratio of a data package that has been successfully transmitted to its next-hop. Generally the *β* of a node reflects how busy the communication channel around the node is.

#### 4.2.2. Scheduling Update

**Figure 5.**
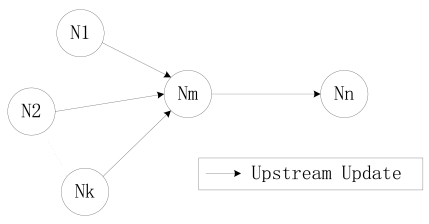
Parameter delivery procedure in O-CDSA.

The parameters of the working state model for each node, the behavior model of its child nodes and the dynamics of the communication channel are all worked out at the SU stage and are used at the next round. At the SU stage, each node only sends its parameter of total transmission time to its parent node, which is referred to the upstream update, as is illustrated in Figure 5. Take note that there is no reverse variables exchanging here, which is the second update process in T-CDSA.

After a node receives the total transmission times of all its child nodes, it calculates the parameters *v* and *m* as follows:

(1) $$v=\sum_{j\in ch}t_{j}/t_{a}$$

(2) $$m=\prod_{j\in ch}\left(1-\overset{t_{j}}{\;}/\underset{t_{a}}{\;}\right)$$

where *ch* is the set of its child node, *t_j_* denotes the total transmission time of node *j* in the last DD stage, and *t_a_* represents the total active time of the node in the last DD stage.

In this paper, in order to avoid the cases that *v* is bigger than 1 or *m* is lower than 0, we set *v* &isin; [0,1] and *m* &isin; [0,1] in case of the severe collision in packet transmission.

The parameter *β* can be computed as below:

(3) $$\beta =\frac{\text{The number of data packets trasmitted successfully}}{\text{The total number of data packets trasmitted}}$$

Equation (3) can be extended to other MAC protocols. For the RTS/CTS based CSMA/CA protocol, the parameter *β* can be obtained by replacing the total number of data packets transmitted and packets successfully transmitted with the number of RTS frames transmitted and the number of ACK frames received.

Our CDSAs are implemented above the MAC layer, so we do not consider the transition probability to state R, state Ix and I_0_, which are jointly decided through negotiations of MAC protocols between the sender and the receiver. Let P(S_i+1_÷S_i_) denote the scheduling probability of SM that a node transmits from state S_i_ to state S_i+1_. By the analysis of Section 4.2.1, to derive the probabilities, we should take into account the ratio of a data packet successfully transmitted by a node to the next hop and the state of its child nodes. Take *p*(*T*÷*R*) = *m* × *β* as an example. In order to successfully transmit a data packet, the precondition for a node to transmit its received data packet depends on the state when all its child nodes stop transmitting and the data packet can be successfully received by its next hop. Therefore, the result of a node's SM can be calculated from Table 1. The SM will instruct operations of the node at the next DD stage, where the first column is the current state, the second column represents the next state and the third column is the state transition probability from the current state to the next state. For a sleep node, it will transfer to state I_0_ after waking up.

As is seen from Table 1, the child nodes have direct influence on the probability to Transmit and the probability to Sleep of its parent node. It is noted that the parent node has indirect influence on its child nodes because the data transmission times of child nodes are related to the probability of their parent node from state I_0_ to state S. For example, if the parent node has an over probability to Sleep state, it will have much more time in Sleep state, leading to more data packets transmission time in its child nodes. Because the parameter *v* of the parent node has much more value, the parent node will probably reduce its time in Sleep state accordingly, this is to say that the child nodes and the parent node affect each other interactively.

### 4.3. Two-step Collaborative Distributed Scheduling Approach

O-CDSA can make a node learn the behavior feature of its child nodes and how busy the communication channel is. Furthermore, we propose the T-CDSA, which also teaches a node to learn the sleeping behavior of its parent node.

#### 4.3.1. Scheduling Process

For T-CDSA, nodes shifting to the Sleep state are not restricted to the condition that their queue be empty. For a CSMA/CA based MAC protocol, the MAC layer will cease the data transmission and discard the data packet if retries for failed transmission attempts equals to a specific threshold [26]. For a RTS/CTS based CSMA/CA protocol, another threshold also restricts the number of retries for RTS frames. Therefore, in T-CDSA, we introduce a new state — Failure (F) — to represent the state that either of these limits is reached. The F state is partly caused by serious collisions in the communication channel. It is also partly caused by the Sleep state of the next-hop, which disables the ability to receive the next-hop. For the former case, the node should consider whether it should re-transmit the data packet again or go to Idle state. For the latter one, it should choose whether it goes to Sleep or not.

**Figure 6.**
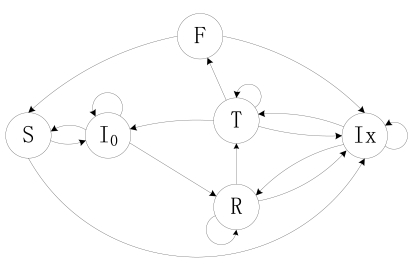
State transition diagram of a node in T-CDSA. where F denotes the state of Failure, S, I_0_, Ix, R and T have the same meaning as mentioned earlier.

When a node sends out a data packet to its next-hop, it will get one of two results accordingly. One result is that it receives the ACK frame, which means the transmission is successful and it can transmit the rest of packets in its buffer or change to another state. Another result is failure to receive the ACK frame and change to the F state. For the latter result, the node can also have two operation choices. One is going to state Ix for the re-transmission attempt or receiving the incoming data packets. Another operation is going to Sleep state because its next hop is in Sleep mode and its child nodes have transmitted all packets in their FIFO. Thus a node goes to Sleep state on the condition that it has transmitted all its data packets in FIFO queue or it is in F state. When the sleep node wakes up, the length of its FIFO queue reflects which state it will transfer to. If the FIFO queue is empty, which means it went to Sleep because it has sent out all of its data packets, it will transfer to the I_0_ state. On the contrary, the node will go to Ix state if it went to sleep due to F state. The new working state diagram is illustrated below in Figure 6.

To schedule states of a node efficiently, the node should estimate the Sleep behavior of its parent node in addition to the communication channel and the behavior of its child nodes. Therefore, we introduce two new variable *g* and *g_p_* to denote the probability of a node and its parent node from F state to Sleep state. The behavior model of child nodes and the dynamics of channel are same as that in Section 4.2.2.

#### 4.3.2. Scheduling Update

**Figure 7.**
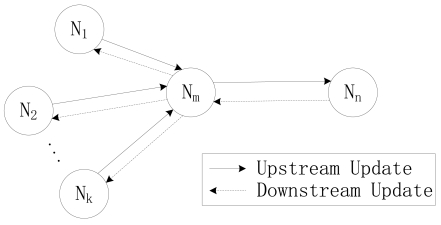
Parameter delivery procedure in T-CDSA. where N_1_ ˜ N_k_ is the child nodes of node N_m_, and node N_n_ is the parent node of node N_m_.

When in T-CDSA, there are three steps for a node to obtain its SM. The three steps are upstream update, downstream update and probability calculation. The upstream update and the downstream update steps are illustrated in Figure 7, which is the reason that we call this approach a two step CDMA.

##### 1) Upstream update

The upstream update step refers to the process that the statistical parameters are transmitted for the upstream direction and the parameters *v* and *m* can be achieved by Equations (1) and (2). This can help a node learn the behavior of its child nodes. This step is same as the updating process of O-CDSA.

##### 2) Downstream update

The downstream update aims to let child nodes learn the sleep behavior of their parent node. After all nodes completed the upstream update, the sink node initiates the downstream update by broadcasting a downstream update packet (DUP). This DUP will be propagated to the leaf node along each route. After a node receives the DUP from its parent node, it extracts the probability information of Sleep state of its parent node and calculates its own parameter *g* - the scheduling probability from F to S as shown below. The *g* is certainly related to the next-hop and its child nodes:

(4) $$g=g_{p}\times (1-v)$$

where *g* represents the scheduling probability from state F to state S, *g_p_* represents the probability of its parent node from state F to state S.

Then the node inserts the parameter *g* into the DUP, and sends the DUP to its child nodes. In this paper, we insert the parameter *g* into the payload of the DUP. Otherwise, if we select the sum of probability from F to S and probability from I_0_ to S as the delivery parameter in DUP, the parameter *g* will probably be bigger than 1. Moreover, let's assume that the WSN system runs into an equilibrium state. Only after all child nodes have transmitted their all data packets and have gone to Sleep, the parent node can choose to Sleep state, i.e. the F state of child nodes is caused by the serious conflict. Only if the next-hop of the parent node goes to sleep earlier than before, it will make the parent node transfer to state F and then move to Sleep state from F state. As the parent node enters to Sleep state earlier than before, it will lead to the F state of child nodes. So it is the probability from F to S that should be inserted in the DUP. And the initial probability value in the DUP is set as follows: the probability of the sink node is 0. And that of the nodes with only one hop distance to the sink node equals to their probability from I_0_ to S.

##### 3) Parameter calculation

The calculation of parameter *β* is same as Equation (3). The new SM of a node can be calculated from Table 2 below (where the first column is the current state, the second column denotes the next state, the third column is the state transition probability value from current state to the next state). The new SM can be derived from the Section 4.3.1 and 4.3.2, which is similar to that of Table 1.

## 5. Analysis of CDSAs

**Figure 8.**
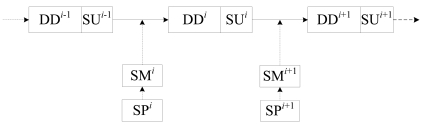
The sequences of SM and SP. where the superscript represents the number of round of a node, SM*^i^* is the scheduling matrix at the *i^th^* round, SP*^i^* is the scheduling parameters at the *i^th^* round, DD*^i^* and SU*^i^* are the data delivery stage and scheduling update stage at the *i^th^* round

In the CDSAs, a new SM of a node is achieved after a round of DD and SU stage, which is illustrated in Figure 8. The SM determines the scheduling probability of the data packets transmission as well as the probability of Sleep state, and determines the work features of a node at the next DD stage. The scheduling parameters (SP), such as *v*, *m*, *g* and *β*, are the fundamental elements of the SM. So it can be used to represent the SM. The sequence of SP decides the work process of a node. Since the next SP depends on the current SP only, the sequences of SP is a Markov process.

In this paper, the WSN is a reliable and loseless network. The remark below will demonstrate that the SP process will be adapted to the workload and the communication channel status of a node.

### 5.1. Adaptability

Each node should sum up *t_a_* - the time in active phrase, *t* – the transmission time of data packets and *β* - the ratio of data packets successfully transmitted at the DD stage. Each node should also calculate the parameter *g* –the scheduling probability from state F to state S in the T-CDSA. Thus we classify the tendency of *t* into three situations and illustrate the adaptability through analyzing the tendency of *t* and *t_a_* as below:

#### 1) *t^n^* > *t^n^*^-1^

Since the nodes generate fixed-size packets at a regular interval, then *t^n^* > *t^n-^*^1^ means either *β^n^* < *β^n-^*^1^ for the same workload as before or more data packets have been transmitted. For the former case, because the parameter *β* reflects the conflict status of the shared channel surrounding the node, the child nodes should also be characterized as a bigger transmission time than before. For the latter case, the child nodes must have transmitted more data packets than before due to a better state of the channel. Therefore, the variable *v* of the node in any case will increase at the next stage by Equation (1), resulting in the decrease of its transmission probability and its probability from I_0_ to S in Table 1 and Table 2. The reduction of the transmission probability will make a lower conflict probability surrounding the node, leading to the decrease of its transmission time *t^n^*^+1^. The decrease of the probability from I_0_ to S will cause the node a lower time in state Sleep and lead to more active time at the next DD stage. For the T-CDSA, *t^n^* > *t^n-^*^1^ also increases the variable *v* of the next-hop. Subsequently the probability of the next-hop from F to S will reduce accordingly. After downstream update, the node itself will have a lower probability from F to S in Table 2. Therefore, the active time at the next DD stage $t_{a}^{n+1}$ will also increase.

#### 2) *t^n^* < *t^n-^*^1^

If *t^n^* < *t^n-^*^1^, we must have either *β^n^* > *β^n-^*^1^ for the same workload as before or less data packets have been transmitted. For the former case, this implies that the successful transmission ratio of data packets for its child nodes is bigger than the previous one. For the latter case, the child nodes must have transmitted less data packets than the last DD stage. Thus, in any case, the random variable *v* will decrease after the scheduling update by Equation (1). This will increase the transmission probability and conflict probability, resulting in an increase in the transmission time *t^n^*^+1^. An increase of variable *v* will also increase the probability from I_0_ to S. Similarly, the probability from state F to state S in T-CDSA will increase. Hence, the node will possess a less active time $t_{a}^{n+1}$ at the next stage.

#### 3) *t^n^* = *t^n-^*^1^

This situation means that the successful transmission ratio of data packets remains stable, which also implies that the successful transmission ratio of data packets for child nodes remains stable. After the scheduling update, the variable *v* will equal to that of the previous one. Therefore, the transmission probability and state scheduling probability of this node from I_0_ to S will not change. Similarly, the variable *v* of the next-hop will keep stable, resulting in a stable probability from state F to state S. That is to say, the probability of state S will not change. Hence, the active time at the next data delivery state $t_{a}^{n+1}$ will keep stable.

**Figure 9.**
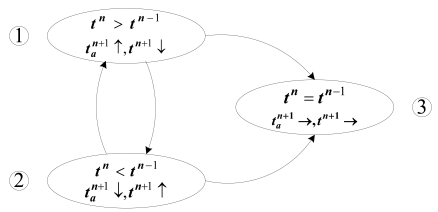
State transition diagram of SP of a node. where ↑ denotes a tendency to rise up at the next DD stage, ↓ denotes a decline tendency at the next DD stage and → represents the stable tendency at the next DD stage.

Figure 9 illustrates the transition relationship. From this figure, we can see that the situation 3 is in an equilibrium state while the situations 1 and 2 above are not stable. Nodes will adapt to the workload and the status of the communication channel, and finally run to the situation 3 after a long time running. When in situation 3, the active time and the data packets transmission time will be stable. Because the active time and data packet transmission time are related to the energy consumption of nodes, the energy consumption of nodes will be subsequently adapted to their workload and communication channel status.

### 5.2. Practicality

The variables to be calculated by each node in the DD stage are no more than four variables, and the variables to be exchanged in the SU stage are no more than two. Moreover, either the SU stage can be arranged in a special time period, or the scheduling parameters can be set into the payload of routing protocol or time synchronizing packets, which are periodic processes in WSN. Therefore, these CDSAs can be applied in the real WSN systems.

## 6. Simulations and Testbed Experiments

To verify our CDSAs, firstly, we carry out some simulations and show that the approaches perform much better than the non-scheduling approach. Secondly, we present results from experiments on a 15-node WSN testbed.

### 6.1. Simulation Settings

To verify our approaches, we consider a network consisting of 200 stationary nodes. Each node has a radio range of 250 m. The nodes are randomly and uniformly distributed in a square area with side length of 2,000 m. The sink node is located at the center of the area. We keep locations of all nodes unchanged.

With reference to the IRIS mote module produced for commercial purposes by Crossbow [27], we set the energy consumption model as 53 mW, 70 mW, 48 mW and 0.033 mW to correspond to the Receive, Transmit, Idle and Sleep states, respectively.

The data communication rate is set as 250 kbps. The application layer of each node is set to generate 100 bytes data simultaneously every 20 seconds. The wake-up time is set every 10 seconds and the scheduling update time is set every 60 seconds.

We use NS2 as simulation environment. The MAC layer adopts the RTS/CTS based CSMA/CA protocol.

In a WSN with dense nodes and synchronous data sampling, serious collisions and energy consumption exist. If the data lossless is not guaranteed, the packet loss rate must be considered as a performance metric. Because the objective of our CDSAs is to reduce energy consumption, we assume packets are lossless. Thus, the sender must re-transmit the data packet if it fails to transmit the packet.

To evaluate our approaches, three groups of experiments are carried out. The first group of experiments adopts the non-scheduling approach, in which only CSMA/CA protocol is applied. This group is used as a benchmark for our two CDSAs. The second group adopts the O-CDSA and the third group employs the T-CDSA. In each group we perform 20 independent experiments, respectively. Each experiment iterates 40 rounds in the program.

The static route is formed at the RD stage. Initially, the sink node broadcasts a route discovery packet (RDP). This RDP is then received by the nodes around the sink node. After a random delay in each node receiving the packet, the node continues broadcasting the RDP. Each node regards the sender of the packet received first as its next-hop and ignores the other RDPs received. The RDP is broadcasted until all the nodes in the network receive it. The static route is formed eventually at the end of the RD stage. The first experiment in each group uses the same seed number at the RD stage, and the rest of experiments use different seed number. Therefore, the static routes in the rest of experiments are different from each other except that the topologies of the first experiment in each group are identical.

### 6.2. Simulation Results

Minimizing energy expenditure always exercises influence over QoS of the WSN [28]. In addition to energy consumption, we study the performance metrics below to evaluate the practicability of approaches in this paper.

1. Energy consumption: The energy consumed in a time period
2. Queue length: The length of FIFO queue
3. Network throughput: The average number of data bytes over the WSN to the sink node
4. Packet delay: The average amount of time that data packets take from the originator to the sink node.

#### 6.2.1. Overview

To obtain a panoramic view of the performances of our CDSAs, firstly we present the performance results of each scheme in Table 3. Each numeric result is the statistic value of 20 simulation experiments in each group. In our simulations, the SP parameters are exchanged through packets at the SU stage instead of in the payload of a routing protocol or other protocol. All nodes use 92.337 W for O-CDSA and 97.628 W for T-CDSA in total at SU stages. The total energy consumption in Table 3 contains the energy consumption used at the SU stages, which is also applied to the Section 6.2.2. As is shown in Table 3, the results show that the two CDSAs can save energy significantly compared with the non-scheduling approach. It can save approximately 38% energy for the O-CDSA and almost 61% energy for the T-CDSA, according to the experimental results. In terms of maximum queue length, it occupies the majority of buffer for O-CDSA, while buffer occupation of T-CDSA is close to that of non-scheduling approach, and in terms of the network throughput, the O-CDSA is slightly slower than other approaches, and the T-CDSA reaches very close to that of non-scheduling. In addition, the packet delay of the O-CDSA is much longer than that of the non-scheduling approach, while the packet delay of T-CDSA is very close to that of non-scheduling approach. In conclusion, the results of T-CDSA are very close to that of non-scheduling in terms of queue length, network throughput and packet delay. With regard to the stability of scheduling approach, most of the standard deviation of performance metrics of T-CDSA is less than that of O-CDSA, i.e., T-CDSA is much more stable than O-CDSA. It is attributed to the sleep scheduling and collision avoidance mechanisms for energy saving and decline in other performances of the CDSAs.

#### 6.2.2. Energy Consumption

**Figure 10.**
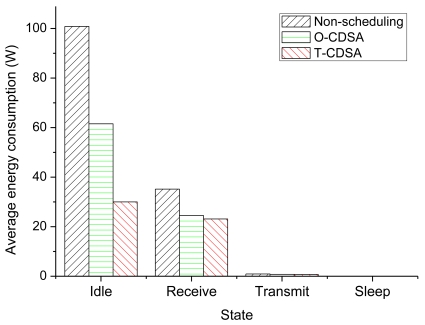
Energy consumption in each state.

To clarify the effects of the two CDSAs on reducing energy consumption, we summarize the energy consumed in each state of the three groups, which are present in Table 4 and Figure 10. Idle state normally consumes the most energy among the node's states in a contention network [1,5]. From Table 4 and Figure 10, we can see that the CDSAs reduce the energy consumption significantly in the Idle and Receive states. The energy reduction is caused by the increase in the Sleep state. Also, the energy consumption in Transmit state is reduced in CDSAs due to the collision avoidance, which helps to reduce total energy consumption. Therefore, the total energy consumption of nodes using our CDSAs is less than the non-scheduling approach. Table 4 and Figure 10 show further that the T-CDSA can reduce energy much more than O-CDSA in the states of Idle, Receive and Transmit.

**Figure 11.**
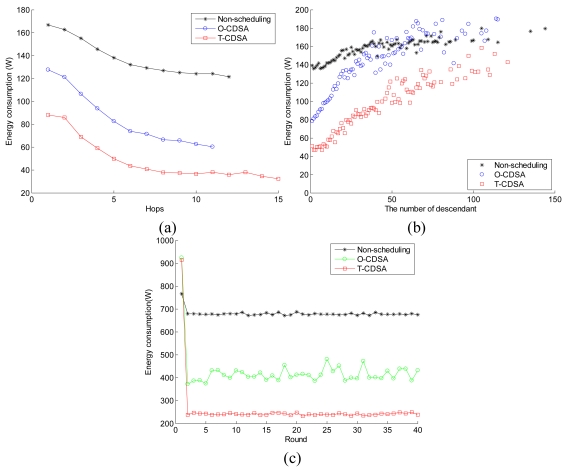
Energy consumption analysis: (a) Average energy consumption versus the hop of nodes. (b) Average energy consumption versus the number of descendants of nodes. (c) Total energy consumption in WSN at each round.

We compare the energy consumption of the three approaches in Figure 11. Figure 11(a) reflects the relationship between the average energy consumption and the nodes' hop. Figure 11(b) shows the relationship between the energy consumption and the number of descendants of nodes. Each data point in Figure 11a,b is the average value of 20 simulation experiments in each group. From Figure 11(a), we find that the energy consumption of a node comes down when it is located close to the sink node, which applies to the non-scheduling, O-CDSA and T-CDSA. For the nodes with the same hop, the energy consumption of O-CDSA is less than that of non-scheduling, and T-CDSA consumes the least. The more descendants a node has, the more workload it has. Therefore Figure 11(b) reflects the relation between the workload of a node and the amount of energy consumption. As is seen from this figure, for all the three schemes, the workload affects the amount of energy consumption, i.e. when the workload increases, the amount of energy consumption increases accordingly. However, the energy dissipation of the CDSAs rises up quicker compared with the non-scheduling scheme. Furthermore, the energy consumption of O-CDSA increases much faster than that of T-CDSA when the workload increases. When the number of descendants is big enough in O-CDSA, its energy consumption will be similar to or a bit higher than that of non-scheduling under the same conditions. This may be caused by re-transmission of data packets in many times when its parent node is in Sleep state. On the contrary, the energy consumption of the T-CDSA is always lower than that of non-scheduling, i.e. the T-CDSA is much stable. From this figure, we can also conclude that the two CDSAs are well adapted to the workload of all the nodes. Figure 11(c) depicts the total energy consumption at each round. And all the data are from the fist experiment in each group. As is seen from the Figure 11(c), T-CDSA can save more energy than the O-CDSA.

#### 6.2.3. Queue Length

**Figure 12.**
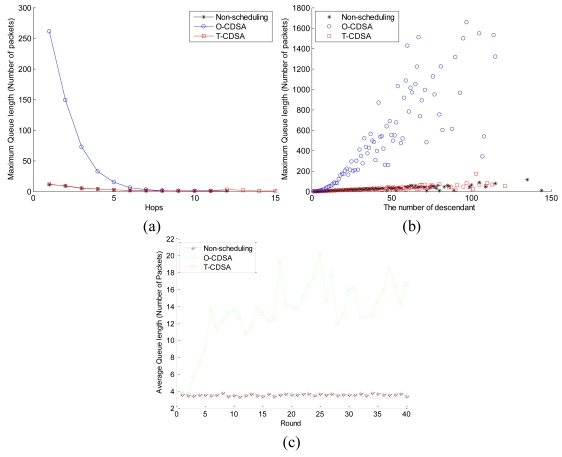
Queue length of the three schemes: (a) Maximum queue length versus the nodes' hop. (b) Maximum queue length versus the number of descendants. (c) Average queue length at each round.

Figure 12 shows the queue length of these three approaches. Each point in Figures 12a,b is the mean value of 20 simulation experiments in each group, and each point in Figure 12(c) is the value from the first experiment in each group. Figure 12(a) reflects the relation between the maximum queue length and the nodes' hop. From Figure 12(a), we can obtain that the maximum queue length falls when the nodes' hops increase. The maximum queue length of the O-CDSA is the biggest value among the three schemes when nodes are located close to the sink node. But it falls down quickly approaching close to that of non-scheduling when the hop is bigger enough. The maximum queue length of the T-CDSA always comes close to that of the non-scheduling scheme. Figure 12(b) reflects the relationship between the maximum queue length and the number of descendants of nodes. From this figure, we can find that the maximum queue length rises up when he number of nodes' descendants increases. In addition, the performance metric of the O-CDSA rises up more quickly than that of the other two schemes. While the maximum queue length of the T-CDSA comes always close to that of non-scheduling approach. Furthermore, Figure 12(c) reflects the average queue length at each round. From Figure 12(c), we can conclude that the average queue length of O-CDSA increases quickly at the initial stage, but keeps below a specific value after several stages. While the average queue length of T-CDSA is below that of non-scheduling in most cases.

#### 6.2.4. Packet Delay

**Figure 13.**
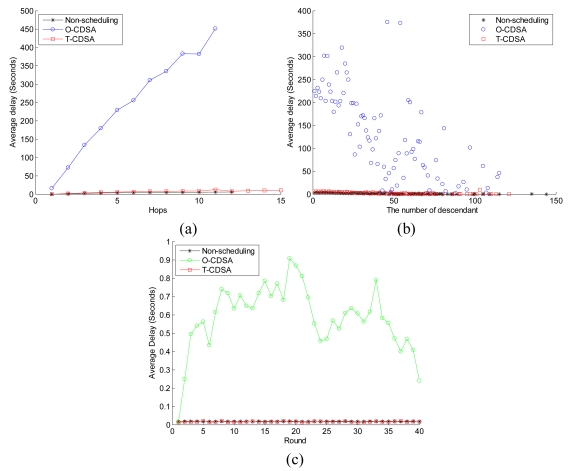
Delay of the three schemes: (a) Average delay versus the nodes' hop. (b) Average delay versus the number of descendants. (c) Average delay at each round.

Figure 13 compares the packet delay between the three schemes. Figure 13(a) represents the relationship between the average delay and the nodes' hop. And Figure 13(b) illustrates the relationship between the average delay and the number of descendants of nodes. Each point in Figure 13(a) and Figure 13(b) is the mean value of 20 simulation experiments in each group. Figure 13(c) illustrates the average delay in each round. Each point in Figure 13(c) is the point of the first experiment in each group. From Figure 13(a), we can obtain that the average packet delay rises up when the nodes' hops increases. And the average delay of O-CDSA rises up much quickly while the average delay of T-CDSA is close to that of the non-scheduling scheme. From Figure 13(b), the average packet delay of the three schemes tends to fall down when the number of descendants of nodes increases. This is more distinct in the O-CDSA. From Figure 13(c), we can get that the average delay of the O-CDSA at each round is always longer than that of others, but will reduce to a lower level for the running rounds.

#### 6.2.5. Simulation Results Summary

In summary, the above simulation results show that the CDSAs we proposed can reduce energy significantly. The CDSAs can effectively reduce energy consumption in the Idle, Receive and Transmit states through the increase of sleep time. In terms of buffer occupancy and delay, O-CDSA is sensitive to the traffic load, and T-CDSA is more robust to the traffic load. So, O-CDSA prefers a light workload application rather than a heavy workload. While T-CDSA can be widely used to many application. In comparison with O-CDSA, T-CDSA presents better network performances and is much more stable. The performance of O-CDSA is not as good as that of T-CDSA due to the absence of estimating Sleep state of the next-hop. However, the T-CDSA requires two updating processes at the SU stage, while O-CDSA requires only one updating process. This shows that O-CDSA is simpler than T-CDSA. Therefore, the SU process of O-CDSA can also be used in a light workload application because it has only one upstream update process. This is to say that the two CDSAs can apply to different applications based on the real application.

### 6.3. Testbed Experiments

**Figure 14.**
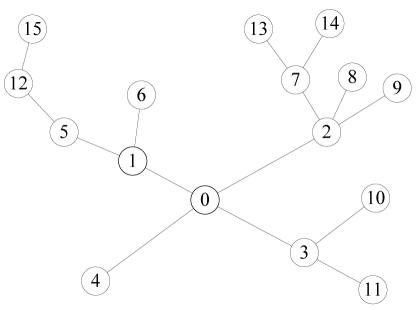
Topology for the WSN testbed experiments.

A series of experiments are carried out to evaluate the performance of the CDSAs on a WSN testbed. The testbed consists of 15 IRIS mote modules [27] in a room where many students and computers are working. The IRIS mote modules uses the Atmel 1281 MCU with program memory 128 KB and SRAM 8 KB. The radio chip of IRIS is IEEE 802.15.4 compliant RF230 radio chip with 2.4G Hz band and 250 kbps data rate. Another 512 KB external serial flash is equipped on the mote for data log. The CDSAs have been implemented in TinyOS 1.x for the motes by Moteworks 2.0, which is a development environment produced by Crossbow. We adopt a static routing structure, as is shown in Figure 14. The CDSAs can also be used in a dynamic routing structure between each routing update process. The base station is located in the center of this room. The CDSAs program occupies about 52,650 bytes in ROM, and 3,878 bytes in RAM. The 3,878 bytes contain 1,100 bytes for queue. The external serial flash is not used in our experiment tests.

Three groups of experiments, similar to the simulation experiments, are carried out. In each group, we perform five independent experiments separately. In each of our experiments, each node originated 200 data packets in total. Each node originates a data packet every 10 seconds with a data payload of 20 bytes and the total packet length 27 bytes. The scheduling update is set every 50 seconds. The battery voltage of each mote node is referred as an indicator of the energy consumption. The voltage is measured by each mote program and transmitted to the sink node as one of the data payload. The experimental results in Table 5 are the statistical values of the five independent experiments. It is noted that the packet delay of each node is not measured in the testbed experiments. Because many mote nodes work as both a data packet originator and a router, it should be in an atomic statement to set data structure of a data packet for concurrent events. But nesC program language in Moteworks does not allow the programmer to do calling commands or signaling events inside atomic statements. Unfortunately getting time value function is put as a command function in Moteworks.

**Figure 15.**
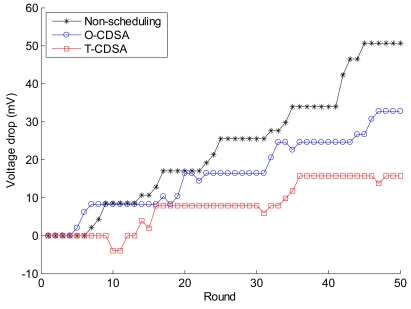
Average voltage drop of node 12 from the three schemes at each round.

As is shown in Table 5, the results are similar to those of the simulation experiments. The voltage drops for Non-scheduling is the quickest, the O-CDSA is second to the Non-scheduling, and the voltage drop for T-CDSA is the slowest among these three approaches. The results prove that the CDSAs can save much more energy than the Non-scheduling. With regard to the stability of scheduling approach, T-CDSA is better than O-CDSA. The results also show that the CDSAs can work well in the testbed experiments. We compare the voltage drop of node 12 from the three approaches in Figure 15. The data are randomly selected from one experiment test in each group. As Figure 15 shows, the voltage of the Non-scheduling approach drops the quickest among the three approaches. For O-CDSA, the voltage drops quicker than the Non-scheduling approach for part of the initial time, but then drops slower than that of Non-scheduling approach. The voltage of T-CDSA drops the slowest among these three approaches. Because the battery is characterized by the recovery capacity effect, the CDSAs can not only make the voltage drop slow, but also increase the voltage sometime after the node goes to sleep. This feature is very interesting and could not be revealed in the simulation results.

## 7. Conclusion and Future Work

Energy efficiency is very important for the lifetime of WSNs. In this paper, we propose two collaborative distributed approaches to reduce energy consumption and prolong the lifetime of WSNs. The adaptability and practicality features are discussed by way of analysis and simulation. The simulation results show that these two approaches can reduce energy consumption effectively. With regard to other performances of WSN, the O-CDSA can apply to lightweight workload applications and the T-CDSA can apply to heavyweight workload applications. The experimental results on a 15-node testbed show that the CDSAs can save energy effectively and are applicable to a real WSN. Our future work will focus on energy efficient routing algorithms and the approaches to balance power consumption for WSNs.

## Figures and Tables

**Table 1.** Scheduling Probabilities of O-CDSA.

**S_i_**	**S_i+1_**	**P(S_i+1_÷S_i_)**
S	I_0_	1
I_0_	S	1 − *v*
R	T	*m* × *β*
I_x_	T	(1 − *v*) × *β*
T	T	(1 − *v*) × *β*

**Table 2.** Scheduling Probabilities of T-CDSA.

**S_i_**	**S_i+1_**	**P(S_i+1_÷S_i_)**
S	I_0_	1
S	I_x_	1
I_0_	S	1 − *v*
R	T	*m* × *β*
Ix	T	(1 − *v*) × *β*
T	T	(1 − *v*) × *β*
F	S	*g_p_* × (1 − *v*)

**Table 3.** Performance comparison of the three schemes.

**Performance Metrics**	**Non-scheduling**	**O-CDSA**	**T-CDSA**
Total energy consumption (W)	27,255.492 (232.407)	16,899.257 (152.045)	10,688.178 (158.359)
Maximum Queue length (Number of Packets)	71.650 (16.989)	946.600 (671.109)	78.700 (27.902)
Network throughput (Bps)	1,194.000 (0.000)	1,051.950 (83.583)	1,194.333 (1.330)
Packet delay (S)	3.801 (0.412)	180.018 (41.854)	5.685 (1.591)

where the statistic value out of parenthesis denotes the mean value of 20 experiments in each group, and the statistic value in parenthesis represents the standard deviation of the 20 experiments.

**Table 4.** Energy consumption in each state.

**State**	**Energy consumption (W)**		

	**Non-scheduling**	**O-CDSA**	**T-CDSA**
Idle	100.867504 (0.788271)	61.526519 (3.212833)	29.996916 (0.181777)
Receive	35.164835 (1.914381)	24.498266 (0.556466)	23.112505 (0.845375)
Transmit	0.929933 (0.042540)	0.776610 (0.017748)	0.728381 (0.056686)
Sleep	0.000000 (0.000000)	0.057428 (0.002256)	0.066978 (0.000280)

where the statistic value out of parenthesis denotes the mean value of 20 experiments in each group, and the statistic value in parenthesis represents the standard deviation of the 20 experiments.

**Table 5.** Performances comparisons of the three schemes in a WSN testbed.

**Performance Metrics**	**Non-scheduling**	**O-CDSA**	**T-CDSA**
Total voltage drop (mV)	770.574 (6.573)	500.620 (5.227)	244.091 (3.861)
Maximum Queue length (Number of Packets)	6.178 (2.390)	10.305 (5.113)	6.207 (3.921)
Network throughput (Bps)	84.000 (0.000)	80.528 (5,202)	83.760 (1.813)

where the statistic value out of parenthesis denotes the mean value of five experiments in each group, and the statistic value in parenthesis represents the standard deviation of the five experiments.
